# Comment on Colletti et al. Prevalence and Management of Cancer of the Rectal Stump after Total Colectomy and Rectal Sparing in Patients with Familial Polyposis: Results from a Registry-Based Study. *Cancers* 2022, *14*, 298

**DOI:** 10.3390/cancers14112650

**Published:** 2022-05-27

**Authors:** Davide Serrano, Irene Feroce, Bernardo Bonanni, Lucio Bertario

**Affiliations:** Division of Cancer Prevention and Genetics, European Institute of Oncology IRCCS, 20141 Milan, Italy; irene.feroce@ieo.it (I.F.); bernardo.bonanni@ieo.it (B.B.)

We recently read the article by Colletti et al. [1]; the large series of familial adenomatous polyposis (FAP) patients is impressive for a mono-institutional cohort. It is very important to develop an effective preventive strategy and to improve the management of rectal stump to reduce rectal cancer incidence and mortality after total colectomy with ileorectal anastomosis (IRA).

The type of prophylactic surgery to choose for FAP patients is a very old and important Gordian Knot [2,3,4]. IRA is often chosen against the ileal pouch–anal anastomosis to preserve a higher quality of life, balancing with the cancer risk of the rectal stump [5].

Recently, to achieve a better personalized medicine treatment, the surgical options for FAP patients have been reconsidered. The IRA role was reevaluated in a larger cohort of patients, with longer follow-up, and, above all, molecular and genotype features [6]. The *APC* gene variants could determine a more aggressive phenotype and be part of the decision for IRA versus a more extensive surgery [7]. It could be very interesting if in Colletti’s article more detailed genotype information could have been provided. They reported that only one-fifth of the patients had the 1309 *APC* pathogenic variant. Besides the frequencies of the genotypes as baseline characteristics, the metachronous rectal cancer could have also been described in more detail in relation to the associated genotype, in particular the above-mentioned variant in relation to cancer characteristics, stage, the timing of the diagnosis, and prognosis. One more issue that could be discussed is whether rectal cancer could be prevented with intensive endoscopic surveillance. In the discussion, the authors stated that all patients adhered to the surveillance protocol. Since surveillance is the main prevention option we could offer for these subjects, it would be important to know the interval between the last negative endoscopy and the rectal cancer diagnosis. Could a more intensive screening could prevent any of these cancers or the mortality from them? The authors reported a median interval of diagnosis of rectal cancer from primary surgery of 13 years, meaning that several of rectal cancer were diagnosed quite early compared with the data of the literature [8,9]. Again, these data evoke questions on the surveillance, or on the biological genotype–phenotype aggressiveness that could have led to an underestimation of the disease at the primary colectomy.

The authors noted that 6% of IRA patients had rectal cancer, apparently very good results compared with the literature [8,9]. Nevertheless, one out of four patients died of rectal cancer, and conservative treatment was possible only in 25 patients (53%). For a more comprehensive analysis, we would like to underline the importance to mention the time from the last rectoscopy and cancer diagnosis, and the number of patients lost at follow-up.

In conclusion, handling rectal stump in FAP patients is still a very complex issue. One main issue is the role of the surveillance and whether, adequately personalized, it can improve cancer incidence and mortality. More details about baseline characteristics and follow-up of a large cohort like the one presented by Colletti et al. can be useful to improve the management of these patients.
